# Fibrillar adhesion with no clusterisation: Functional significance of material gradient along adhesive setae of insects

**DOI:** 10.3762/bjnano.5.95

**Published:** 2014-06-12

**Authors:** Stanislav N Gorb, Alexander E Filippov

**Affiliations:** 1 Department Functional Morphology and Biomechanics, Zoological Institute of the Kiel University, Am Botanischen Garten 1–9, D-24098 Kiel, Germany; 2 Donetsk Institute for Physics and Engineering, National Academy of Sciences of Ukraine, Donetsk, Ukraine

**Keywords:** adhesion, attachment, biomechanics, computer modelling, cuticle, locomotion, material, surface

## Abstract

It has been recently demonstrated that adhesive tarsal setae of beetles possess material gradients along their length. These gradients presumably represent an evolutionary optimization enhancing the adaptation to rough surfaces while simultaneously preventing clusterisation of the setae by lateral collapse. The numerical experiment of the present study has clearly demonstrated that gradient-bearing fibers with short soft tips and stiff bases have greater advantage in maximizing adhesion and minimizing clusterisation in multiple attachment–detachment cycles, if compared to the fibers with longer soft tips on the stiff bases and fibers with stiff tips on the soft bases. This study not only manifests the crucial role of gradients in material properties along the setae in beetle fibrillar adhesive system, but predicts that similar gradients must have been convergently evolved in various lineages of arthropods.

## Introduction

The contact formation of insect adhesive pads on various substrates depends on the pad ability to adapt to different surface topographies. The quality of contact may be increased due to the presence of specific micro- and nanostructures [1–5]. Crack trapping mechanisms in adhesive systems with multiple contacts provide advantages in attachment on rough substrates [6]. Also hierarchical organization of insect pad structures enables formation of multiple contacts that contribute to an enhancement of overall length of the total peeling line [7].

We have recently shown that thin tape-like contact tips of hairs (setae) in combination with applied shear force lead to the formation of maximal real contact area without slippage within the contact [8]. Due to this reason, the material flexibility is important for contact formation of adhesive pads. Flexible materials may generate large contact area between the pad and substrate at minimal normal load. On the other hand, elongated structures, made of too flexible materials, have low mechanical stability [9]: insect setae made of too soft material can buckle and collapse resulting in so called clusterisation/condensation [10–11]. Due to such clusterisation, functional advantage from multiple adhesive contacts may strongly decrease. That is why, material properties of insect adhesive setae represent an optimization problem, which is solved in the course of biological evolution by the presence of gradients of thickness and mechanical properties. Thickness gradients of insect setae are well known in various adhesive setae due to numerous scanning electron microscopy studies [1]. Recently, we presented the combined study on the material structure and local mechanical properties in tarsal setae of the beetle *Coccinella septempunctata* and demonstrated the presence of a material gradient at the level of each single seta [12].

Setal elasticity modulus, probed by atomic force microscope (AFM), ranges from 1.2 MPa at the tip [12] to 6.8 GPa at the base. At the setal tip, we revealed the rubber-like protein resilin in rather high concentrations [13–14], whereas at the base of the seta the sclerotised cuticle is dominating. Between tip and the base, there is a gradient of material composition revealed by confocal laser scanning microscopy (CLSM). This gradient is hypothesized to be an evolutionary optimization enhancing adaptation of adhesive pads to rough surfaces, while simultaneously preventing setal clusterisation. Such an optimisation presumably increases the performance of the adhesive system in general. However, this hypothesis is difficult to prove experimentally using native biological specimens. That is why we decided to test it by the numerical simulation, which is the main aim of the present study.

In this paper we ask following questions:

1. Does the presence of the material gradient along the setae contribute to the proper contact formation?
2. Which particular gradient reduces clusterisation of setae?

## Results and Discussion

### Structure and material properties of biological system

**Figure 1:**
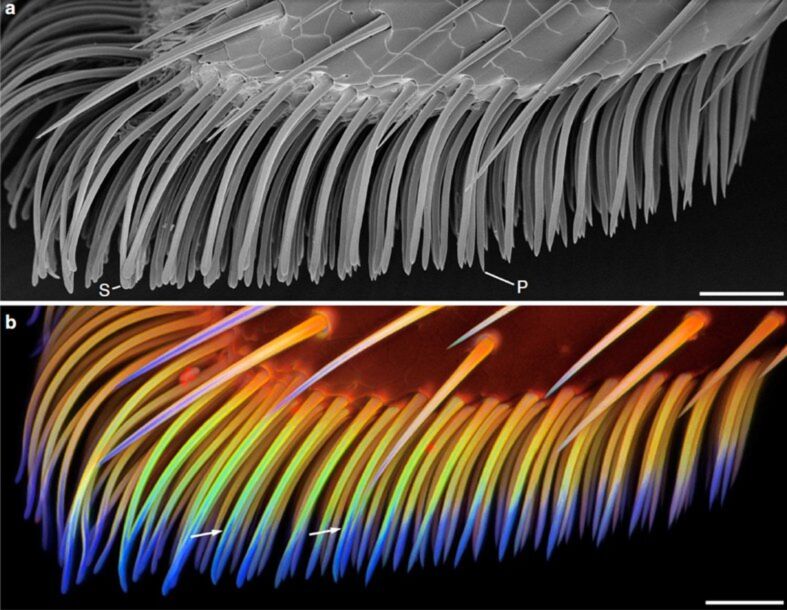
Morphology and material composition of adhesive tarsal setae. Ventral part of the second adhesive pad of a foreleg of a female *Coccinella septempunctata*, lateral view. (a) Scanning electron micrograph (the specimen was dried using 1-propanol). (b) CLSM maximum intensity projection showing an overlay of the four different autofluorescences mentioned in the text. The arrows indicate the dorso-ventral material gradient in exemplary setae. S, exemplary spatula-like seta; P, exemplary seta with a pointed tip. Scale bars, 25 µm. From [12] (Nature Publishing Group).

Previous CLSM analysis of the setal tips has clearly demonstrated the presence of the rubber-like protein resilin in rather high concentrations [12,15]. Both central and proximal parts of the setae were dominated by green, yellow and red autofluorescences due to the presence of other presumably sclerotised proteins and very likely chitin (Figure 1). Between the resilin-dominated distal part and more sclerotised basal part of the seta, a rather pronounced longitudinal gradient of material composition was revealed. AFM-nanoindentation experiments have revealed rather low elasticity modulus at the setal tip (1.2 ± 0.3 MPa), but the high one at the setal base (2.43 ± 1.9 GPa) [12]. This information about the gradients of material properties in real beetle setae was used in the numerical model presented below.

### Numerical model

In principle, to model mechanics of the setae a classical beam theory can be applied. However, for long array of the beams it needs in extremely time consuming numerical calculation. To avoid it we apply here minimalistic, but quite realistic model, which was proposed for the same system few years ago and described in details in the paper [16]. Here we adapt the model to include gradient material properties of insect setae. The model includes following elements. An array of initially parallel fibers attached to a hard planar base. Stiffness of the fibers *F_elastic_* is continuously varied along their length and can be changed from very soft one to much stiffer or even almost rigid one (but still with some degree of flexibility). Longitudinal 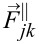 and transversal 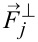 stiffness of the fibers are simulated by the following interaction between the segments 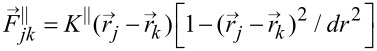, and 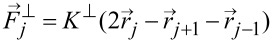.

Here we limit ourselves by two-dimensional model, where values 
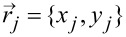
 are the coordinates of the beginning of the segment *j*; *k* = *j* ± 1. Longitudinal force, 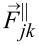, is described by a two-minima potential, which tends to keep a distance between the points 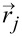 and 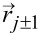 close to the equilibrium length of the segment *dr*. Transversal force, 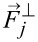 keeps 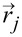 close to the mean value 
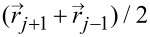
 between its nearest neighbors, and tends to hold the angle between the neighboring segments close to 180°.

The ends of the fibers are attracted to the surface by a sum of molecular and capillary forces. For the sake of simplicity we simulate it by the gradient of Morse potential *U_vdW_*(*r*) = *U*_0_(1 – exp(−*r*/*r*_0_))^2^, where *r* is a distance between the end of fiber and surface, with physically reasonable amplitude *U*_0_ = 10 nN·nm and the minimum located at the distance *r*_0_ = 0.01 µm from the surface [17–18].

Rigid surface of the substrate, where the fibers attach to, has semi-fractal structure with given Fourier spectrum and amplitude of roughness [8]. It can be simulated similar to the approach we previously used in [8] by the self-affine fractal surface given by real part of 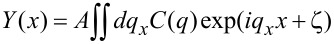 with scaling spectral density. Here *A* is amplitude of surface roughness, *i* is imaginary unit, *q_x_* are Fourier components along *x* direction, and ζ is a random phase.

Details of the generation procedure for the profile *Y*(*x*) have been described in a number of previous papers [19–20]. In the current literature [21] it is accepted that majority of physical surfaces have scale-invariant spectrum *C*(*q*) = 1/*q*^β^ with exponent β ≈ 0.9. The amplitude of the numerical “surface” is taken to be comparable with the radius of van der Waals interaction *A* = *r*_0_.

Soft parts of every fiber, which normally are physically thin and flexible, interact with corresponding regions of other fibers of the array. Since for the majority of studied biological fibrillar adhesive systems, there is no evidence that seta–substrate and seta–seta interactions are different, we assumed that interaction force has the same (van der Waals) origin as their attraction to the hard wall. Assumptions similar to this have been also previously taken by other authors for their models [10–11]. Due to this, it is natural to take it in the same form *U_interact_*(*r_jk_*) = *U*_0_(1–exp(–*r_jk_*/*r*_0_))^2^ with comparable characteristic parameters *U*_0_,*r*_0_. For simplicity of the model, we reduce mutual interaction of the fibers by the interaction of the nearest neighbors: 
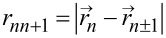
.

For studied problem, one can neglect effects of inertia and treat the system as over-damped. In this approximation, differential equation of motion does not contain second time derivative and can be formally written in the form 
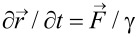
, where γ is dissipative constant and force accumulates all above interactions 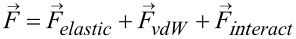. As usually, corresponding components of the forces in the equations of motion are equal to the derivatives: 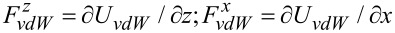, 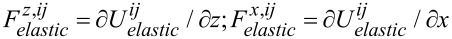 and 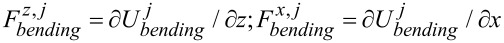. Below we ‘a-posteriori’ normalize γ^−1^ to get typical relaxation times of the system (around 10 ms).

**Figure 2:**
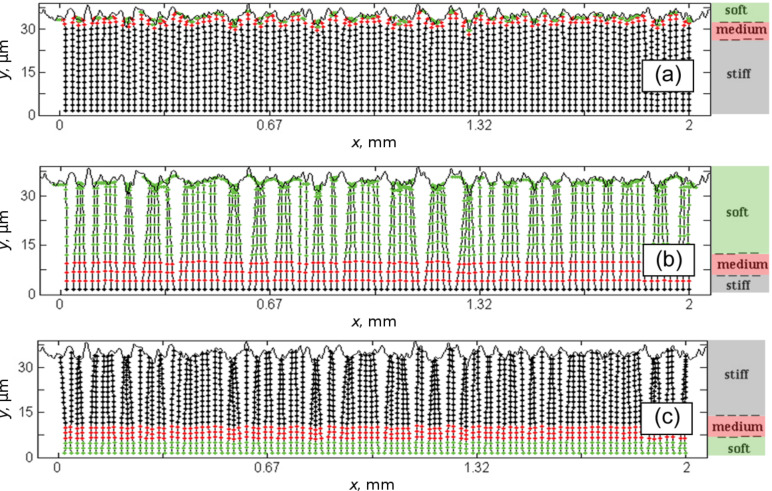
Typical configurations of the filamentary structure (setal array) attached to the stiff support (below) in adhesive contact with random fractal surface (above). This numerical model was used to mimic biological setal arrays shown in Figure 1. Three types of fibers, (1) stiff fibers with short elastic ends, (2) long elastic fibers connected to the base by short stiff roots, and (3) stiff fibers with soft elastic segments near the base are shown in subplots (a), (b) and (c), respectively. Different stiffness of segments is conditionally shown by circles with different colours. Stiff, medium and soft segments are marked by black, red and green circles, respectively.

Conceptual structure of the model is illustrated in Figure 2. Rigid surface of the substrate is shown by upper solid curves. In order to understand the potential functional role of the material gradients found in beetles [12], we study three different kinds of fiber arrays: (a) long stiff fibers with short elastic ends; (b) long elastic fibers connected to the basal plate by the short hard roots; (c) relatively stiff fibers with short soft elastic filaments connected to the base. All these variants are shown in subplots (a), (b) and (c) respectively.

In all the cases the stiffness of the fibers is continuously varied along vertical coordinate. To simulate it we apply smooth step function Θ(*y*) = 1/[1 + exp(−(*y* – *y*_0_)/Δ)] with regulated position of bend *y*_0_ and width Δ. This function tends to unit, when *y* << *y*_0_, and gradually goes to zero in the opposite limit. This allows modeling all above mentioned cases in common approach. Stiffness previously experimentally estimated for insect adhesive setae are as follows: 1.31 N·m^−1^ (fly *Calliphora vicina* [22]), 0.192–0.693 N·m^−1^ (beetle *Gastrophysa viridula* [23]).

To illustrate different stiffness of fiber segments shown in the Figure 2, we formally divided the stiffness into three regions: (1) close to the maximal stiffness, (2) less than half of the maximal stiffness (a region around *y*_0_ with the width Δ), and (3) less than 0.1 of the maximal stiffness. These parts are conditionally shown in the plots by different colours. Stiff, medium and soft segments are marked by black, red and green circles respectively. It is important to mention that our model is certainly limited. It does not account for plastic deformations, geometrical nonlinearity due to large deformations or friction effects. It is focused only on study of the effect of stiffness gradient on contact adhesion problem.

Our numerical procedure is organized as follows. We take originally unperturbed arrays of parallel fibers attached to the horizontal hard base, bring them into contact with numerically generated rigid fractal surface and solve numerically differential equations of motion by standard procedure of Matlab software. The fibers distort due to interaction between them and surface as well as due to their mutual interaction with the neighbors. Many of fibers are attracted to the same individual asperities of the surface. This attraction enhances their mutual interaction in contrast to original unperturbed state.

One can record time-depending distortions of the fibers as well as variation of the interaction forces, to control the process of contact formation and stop it, when the system reaches certain stationary configuration. After this, we can remove rigid substrate surface and allow the system to relax spontaneously to some new stationary state.

Many of the fibers, which were preliminary attracted to the same asperities of the surface, still strongly interact and remain close one to another, collecting into local clusters. Mutual attraction between the fibers competes with the elastic forces inside the fibers which try to return them to the straight position and the whole array as well to its original parallel-organized structure. Further scenario of setal arrangement development certainly depends on the relationship between these forces and their spatial distribution. In some cases, structure can return back to the original state, but in some cases it can not. If it is so, the fibers remain collected into strongly confined bunches (so-called clustering/condensation phenomenon).

**Figure 3:**
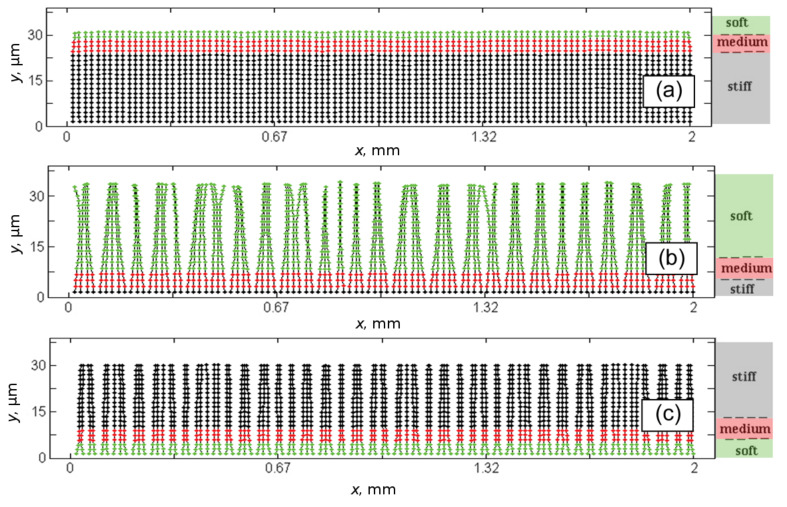
The same system as presented in Figure 2 shown after detachment from the fractal surface and sufficiently long relaxation to the static state. The difference between strongly clustered systems having either long elastic (b) or long stiff filaments (c) and the system with short soft ends (a), which practically returns back to its original configuration, is clearly seen. See also complementary movies 1, 2, 3 (Supporting Information File 1), which correspond to the cases (a), (b), (c), respectively.

This phenomenon is very important from the practical point of view, because the clustered system is not ready to attach efficiently to every new surface during next contact events. That is why, in the present work, we mainly concentrated on the study of this effect. Qualitative results related to the clustering are summarized in Figure 3, where the same systems shown in Figure 2 are presented after their detachment from the surface and sufficiently long transient period of relaxation to the static state.

It is clearly seen that in contrast to strongly clustered systems with long flexible (Figure 3b) or long hard filaments (Figure 3c), the system with long hard filaments having short flexible ends (Figure 3a) practically returns back to its original configuration. This observation leads to a very important question. To get complete return to the original state after relaxation, it is important to have quite short flexible ends of the fibers in contrast to their complete length, but may be strong deformation of these ends in attached state is not enough to produce sufficiently strong attachment force?

**Figure 4:**
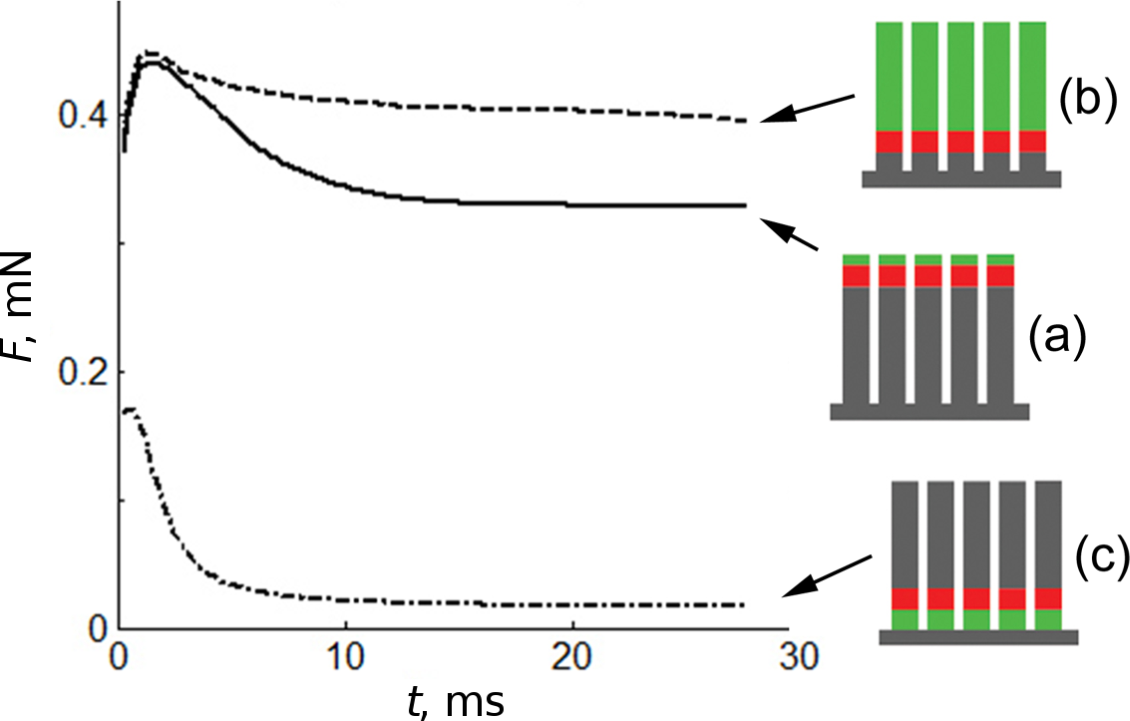
Time depending vertical forces developed during attachment of initially unperturbed systems to the hard surface. Solid, dashed and dash-dotted lines correspond to the cases (a), (b) and (c) of previous figures, respectively.

To compare attraction forces in all the cases (a)–(c), we performed their accumulation over all contacting segments during entire time interval of the attachment (Figure 4). Let us remind that first stage (attachment) of our numerical experiment is organized as follows. We take originally unperturbed arrays of parallel fibers and put them on the horizontal rigid base, at fixed distance from numerically generated rigid fractal surface. The fibers adapt to the surface. During this process the force between them and surface changes and we record its time dependence while it reaches stationary asymptotic value. Because rigid horizontal base is fixed the force never falls down to zero, but tends a constant value depending on the case (a)–(b). It is seen from the Figure 4 that maximal forces in the cases (a) and (b) are comparable. Moreover, the potential barrier (the difference between maximum of force at the beginning and its minimum, which system gets after good adaptation to the rigid surface) is even higher in the case (a). Qualitatively this effect appears, because flexible filaments are too long in the case (b). Last case (c) with long hard filaments rotating around their flexible roots, cannot perfectly adapt to the surface. As a result, maximum of the attachment force here remains much lower than in two previous cases (a) and (b).

**Figure 5:**
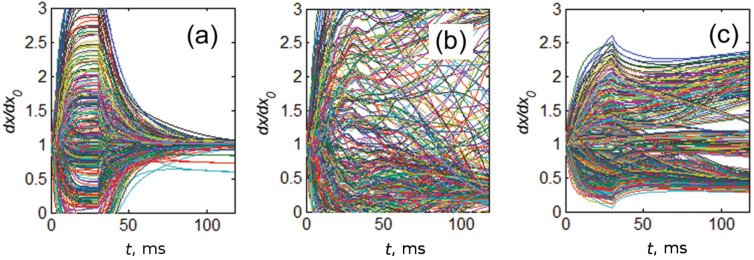
Time evolution of arrays {*dx_j_*} of distances *j* = 1,2,…*N_x_* between ends of nearest neighbors *dx_j_* = *x_j_*_+1_ − *x_j_* during single attachment–detachment cycle for the same cases (a)–(c) as before. All the distances are normalized to the distance of original unperturbed periodic system: *dx_j_* = *dx*_0_ at *t* = 0. Each line in the plots corresponds to a time-depending distance between one pair of the neighbors *dx_j_* = *x_j_*_+1_ − *x_j_*. In attached state, all the filaments tend to a configuration, which represents certain compromise between stiffness, adhesion to the surface and mutual interaction of the fibers. After detachment, the system relaxes to asymptotic configuration corresponding to a compromise between the stiffness and mutual interaction of the fibers only.

To accumulate time-dependent information about deformations of the fibers we calculate array {*dx_j_*}, *j* = 1,2,…*N_x_* of the distances between contact ends of the nearest neighbors *dx_j_* = *x_j_*_+1_ − *x_j_*. Let us note that we are using *dx_j_* for small but finite distances (not differential). We use this notation to conserve coincidence with all our previous publications using the same or close models and hope, it will not cause any misunderstanding. Time evolution of every such array during complete attachment–detachment cycles for all (a)–(c) cases is shown in Figure 5. Each line in the plots corresponds to one particular time-depending distance between a pair of the closest neighbors *dx_j_* = *x_j_*_+1_ − *x_j_*. All these distances are normalized to the distance of original unperturbed periodic system, so *dx_j_* = 1 at *t* = 0.

The history of the process is clearly seen from the plots. When some fibers are attracted to the same asperities of the surface and form the clusters, the distance between their ends goes to zero *dx_j_* = *x_j_*_+1_ − *x_j_* → 0. At the same time, the distance between the fibers from different clusters generally grows. This distance must correlate with a characteristic distance between the asperities, but it remains random for random fractal surface. Finally, attached configuration delivers a complex compromise between: (1) stiffness of the fibers; (2) fractal structure of the surface; (3) strengths of all the interactions.

When the surface is removed, the system of fiber array relaxes to new final configuration which is driven by a compromise between stiffness and mutual interaction of the fibers only. If stiffness dominates, the system can return to the original unperturbed state. Time-depending history of this process is clearly recorded in the subplot Figure 5a. It is interesting to note that stiff fibers of case (c), having strong elastic energy cannot completely return back to initial state. They remain glued by their top ends.

**Figure 6:**
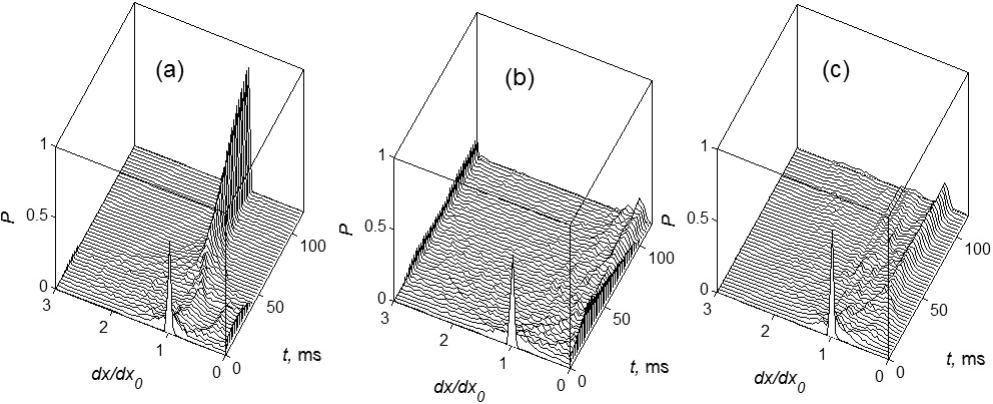
Statistical analysis of the plots presented in Figure 5. The sequences of the histograms show time evolution of the probability *P* = *P*(*dx*) to find a particular value of the distance *dx_j_* = *x_j_*_+1_ − *x_j_* between nearest fibers. The cases (a)–(c) are the same as above. Starting from unperturbed configuration (initial peak of probability around *dx_j_* = *dx*_0_) all the systems evolve to the smooth distributions *P*(*dx*). In clustered attached state (see cases (a) and (b)) the probabilities have well pronounced maximums at *dx* ≈ 0. After detachment from the surface all the systems tend to the distributions *P*(*dx*), which perfectly agree with the observed final states shown in Figure 3 and Figure 5.

To analyze the results statistically one can calculate histograms of the probability *P* = *P*(*dx*) to find a particular value of the distance *dx_j_* = *x_j_*_+1_ − *x_j_* between nearest fibers. It is done for a sequence of discrete time steps and summarized in Figure 6. The cases (a)–(c) in this figure are the same as above.

These data make information presented in Figure 5 clearer. Initial peak of the probability around *dx* = 1 corresponds to almost unperturbed configuration at small time, just the first contact with the surface. As time goes by around 25 ms all three types of systems deform their filaments into the configurations with smooth distribution *P*(*dx*). It means that different distances *dx_j_* = *x_j_*_+1_ − *x_j_* appear with comparable probabilities. With the time, in systems with soft ends ((a) and (b)), many fibers are attracted to the same asperities of the surface. As result the probabilities get well pronounced maximums near *dx* ≈ 0. After detachment all the systems tend to asymptotic probability distributions which perfectly agree with the observed final configurations shown in Figure 3 and Figure 5.

### Biological significance

Pure bulk materials are absent in biology: biological materials are always composites. Also material gradients are well known in biological systems, where particular change in composition of different bulk materials along a biological structure may lead to novel and often unexpected properties. This has been previously shown for insect cuticle [24–25], snake skin [26], human teeth [27–28], and other biological composites.

The gradients have been also recently reported for smooth attachment devices of insects [29]. Interestingly, the gradients in smooth pads of locusts and bushcrickets are different from gradients reported in hairy pads of ladybird beetles [12]. Smooth adhesive pads consist of a softer core covered by a stiffer layer, whereas hairy pads have opposite arrangement: stiffer bases combined with softer distal part. Both types of gradients combine conformability to the surface roughness of the substrate and resistance to the environment.

The opposite directionality of gradients can be well explained by difference in pad architecture. Smooth pads consist of branching rods or cellular foams, which in combination with fluid-filled spaces between solid structures hold the shape of the pad. This principle is combined with the presence of a relatively stiff superficial layer that terminates the fibers. The layer keeps the distance between tips of fibers at some constant value (and in species living in arid environments protects the pad from desiccation) [29–30]. In the hairy pads, adhesive setae are not terminated by continuous layer and can potentially buckle and cluster together [10–11]. As strong degree of clusterisation leads to the decrease of functional advantages from multiple contacts [7], this is reduced by the presence of gradients of thickness [1] and mechanical properties [12].

Whereas disadvantages of purely stiff and purely soft fiber arrays are intuitively clear, it is difficult to judge about the advantages of various gradients from the fiber base to the fiber tip (soft-to-stiff/downstream and stiff-to-soft/upstream). The numerical experiment of the present study has clearly demonstrated that gradient-bearing fibers with short soft tips and stiff gradients (short upstream gradient) has greater advantage in maximizing adhesion and minimizing clusterisation in multiple attachment-detachment cycles, if compared to the fibers with longer soft tips on the stiff bases (long upstream gradient) and fibers with stiff tips on the soft bases (downstream gradient). Such short upstream gradients were recently described in beetles [12], however, we can predict that similar gradients must have been convergently evolved in various lineages of arthropods.

## Supporting Information

**Movie 1:** Behaviour of the model array of setae/fibers, which have short soft ends and stiff bases during attachment-detachment cycle (a). Different stiffness of the segments of fibers is conditionally shown by different colors. Stiff, medium and soft segments are marked by black, red and green circles respectively. The subplots in the bottom (from left to right) show time dependent vertical force, evolution of the array of distances *dx_j_* = *x_j_*_+1_ − *x_j_* between contact ends of nearest neighbors and instant histogram *P*(*dx*) of the distribution of these distances. It is seen directly from the movie, how the system deforms near the surface and how it gradually returns back to the original state after detachment.

**Movie 2:** The same as Movie 1 for the array of long soft fibers. The colors and subplots are the same as those in the Movie 1. In contrast to the previous case, this system cannot overcome strong deformations of mutually glued filaments and does not return to the original unperturbed state.

**Movie 3:** The same as the previous Movies 1 and 2 for hard fibers softly connected with the bottom plate by few soft intermediate segments. Despite of stiffness of the filaments the structure is still able to adapt to the surface due to fiber rotation around their soft parts. As result, system gets quite satisfactory attachment to the rough surface, but it practically does not return back to the initial unperturbed state after detachment.

File 1 Movies 1–3.
